# The "Æsthetics of Suicide."

**Published:** 1862-04

**Authors:** 


### THE "ÆSTHETICS OF SUICIDE."

In the last number of the *American Journal of the Medical Sciences*, Dr. S. H. Dickson, Professor of the Practice of Medicine in Jefferson Medical College, Philadelphia, dissents (see Art. VI.) from the tendency of an article which appeared in the October number of this Journal under the title of "*The Æsthetics of Suicide.*" Dr. Dickson erroneously attributes this article to the pen of the Editor; and the author is wishful to correct a misapprehension into which he thinks Dr. Dickson has fallen. He says:—

"Dr. Dickson objects that the article referred to is 'unjust and injurious in its pervading suggestion of a relationship assumed to exist between the suicidal disposition in the mind, and a moral degeneracy in the character. This assumption I believe to be entirely unfounded.' Dr. Dickson then cites several cases of suicide for the purpose of showing that suicide, 'however deeply to be deplored, however earnestly to be reprobated and forbidden, is not necessarily the result of any moral degeneracy, nor essentially associated with any imputation of vice or crime.' The latter proposition, I would remark, has no necessary connexion with the former, and I submit that the supposition upon which it is based forms no part of, and is not to be derived, except under misapprehension, from, an article, the object of which was intended to indicate in what manner, according to the author's opinion, a certain æsthetical method of treating suicide, in vogue in Europe, might, and most probably did, foster the act of self-murder. I had wished to convey the notion that this æstheticism of suicide was a highly influential cause *predisposing* to the commission of self-murder—an opinion which I believe is entirely in accordance with the views expressed by yourself [the Editor] in the 'Anatomy of Suicide,' and which is amply supported by the observations and conclusions of the most trustworthy and recent writers on the subject, as, for example, Brierre de Boismont and Lisle. I venture to suggest, indeed, that Dr. Dickson has misapprehended the object of my article so far as the immediate motives leading to the act of suicide are concerned. These, in fact, formed no part of my subject, which was limited to a consideration of certain of those circumstances which most tend to induce that state of mind in which the proximate causes, the immediate motives of suicide, prove influential in inducing the act. If the proximate,—the determining causes of suicide proved influential absolutely, without reference to some pre-existing mental state, instead of relatively with reference to such an assumed state, suicide would be the rule in ordinary life, and not the exception. That 'a moral degeneracy in the character' has a most important influence in fostering a disposition of mind favourable to the commission of suicide, by enfeebling the great moral checks to that act, and thus predisposing to self-murder, I undoubtedly hold; and the main object of my article was to show in what manner a class of literature, characterised in an especial way by an apotheosis of vice, contributed to this end. This question is of such great importance in reference to suicide, that I will ask your permission to deal with it again in the next number of the Journal—confining myself to such portions of my argument as I then omitted, partly from a desire not to intrude too much upon your space, partly from the belief that it was not necessary to state at large on what grounds I adopted certain primary assumptions which had been long received, as I thought upon the most sufficient and conclusive data, by the best authors on the subject. In the meanwhile, however, unwilling that it should be supposed, by any doubting readers, for a moment longer than necessary, that the question of the relationship of vice and immorality to suicide is one of assumption only, I quote the following observations from Brierre de Boismont's great work on '*Suicide*,' (p. 87). Of 4595 cases of suicide examined by that authority, the *morality* was *good* in 1945, *bad* in 1454, *unknown* in 1196. Thus in upwards of *forty per cent.* (42·7) of the cases in which the actual moral condition was ascertained this was thus found to be bad, the individuals having been drunkards, gamblers, or thieves, or lived in concubinage or adultery. The drunkards formed a fourth part of this class, gamblers a sixty-seventh, and thieves a seventieth; those who lived in adultery a nineteenth, and in concubinage a fifth. 'But,' observes M. B. de Boismont, 'the proportion of those individuals leading a regular life, and who have attempted to shorten their days, diminishes singularly, if it be recalled to mind that there are among them a considerable number of lunatics, weak-minded, and exalted spirits, hypochondriacs, sick, and persons reduced to poverty and misery. It is true then that the vices and passions exercise an unfavourable influence upon our acts and determinations, and that those who abandon themselves to them have in prospect poverty, a prison, sickness, insanity, and suicide.'

"M. B. de Boismont's observations and remarks refer to *overt* immorality; but do drunkenness, concubinage, adultery, gambling, and crime, include all the phases of a 'moral degeneracy of character?' Is not that state of mind equally a degeneracy, which, while inducing an individual to give due heed to all the conventional rules of morality, yet ignores and contemns those higher religious considerations which, I hold, alone makes a self-sought death an evil to which none experienced in this life can compare? Does not the philosophy of the great Stagirite shame many a Christian writer? 'The suicide,' he said, 'does not undergo death because it is honourable, but in order *to avoid a greater evil*.'"

